# Vitamin E δ-Tocotrienol Induces p27^Kip1^-Dependent Cell-Cycle Arrest in Pancreatic Cancer Cells via an E2F-1-Dependent Mechanism

**DOI:** 10.1371/journal.pone.0052526

**Published:** 2013-02-05

**Authors:** Pamela J. Hodul, Yanbin Dong, Kazim Husain, Jose M. Pimiento, Jiandong Chen, Anying Zhang, Rony Francois, Warren J. Pledger, Domenico Coppola, Said M. Sebti, Dung-Tsa Chen, Mokenge P. Malafa

**Affiliations:** 1 Department of Gastrointestinal Oncology, H. Lee Moffitt Cancer Center and Research Institute, Tampa, Florida, United States of America; 2 Department of Molecular Oncology, H. Lee Moffitt Cancer Center and Research Institute, Tampa, Florida, United States of America; 3 Department of Pathology, H. Lee Moffitt Cancer Center and Research Institute, Tampa, Florida, United States of America; 4 Department of Drug Discovery, H. Lee Moffitt Cancer Center and Research Institute, Tampa, Florida, United States of America; 5 Biostatistics Core, H. Lee Moffitt Cancer Center and Research Institute, Tampa, Florida, United States of America; 6 School of Life Science and Technology, University of Electronic Science and Technology of China, Chengdu, People's Republic of China; 7 College of Medicine, University of Florida, Gainesville, Florida, United States of America; Vanderbilt University Medical Center, United States of America

## Abstract

Vitamin E δ-tocotrienol has been shown to have antitumor activity, but the precise molecular mechanism by which it inhibits the proliferation of cancer cells remains unclear. Here, we demonstrated that δ-tocotrienol exerted significant cell growth inhibition pancreatic ductal cancer (PDCA) cells without affecting normal human pancreatic ductal epithelial cell growth. We also showed that δ-tocotrienol-induced growth inhibition occurred concomitantly with G_1_ cell-cycle arrest and increased p27^Kip1^ nuclear accumulation. This finding is significant considering that loss of nuclear p27^Kip1^ expression is a well-established adverse prognostic factor in PDCA. Furthermore, δ-tocotrienol inactivated RAF-MEK-ERK signaling, a pathway known to suppress p27^Kip1^ expression. To determine whether p27^Kip1^ induction is required for δ-tocotrienol inhibition of PDCA cell proliferation, we stably silenced the *CDKN1B* gene, encoding p27^Kip1^, in MIAPaCa-2 PDCA cells and demonstrated that p27^Kip1^ silencing suppressed cell-cycle arrest induced by δ-tocotrienol. Furthermore, δ-tocotrienol induced p27^Kip1^ mRNA expression but not its protein degradation. p27^Kip1^ gene promoter activity was induced by δ-tocotrienol through the promoter's E2F-1 binding site, and this activity was attenuated by E2F-1 depletion using E2F-1 small interfering RNA. Finally, decreased proliferation, mediated by Ki67 and p27^Kip1^ expression by δ-tocotrienol, was confirmed *in vivo* in a nude mouse xenograft pancreatic cancer model. Our findings reveal a new mechanism, dependent on p27^Kip1^ induction, by which δ-tocotrienol can inhibit proliferation in PDCA cells, providing a new rationale for p27^Kip1^ as a biomarker for δ-tocotrienol efficacy in pancreatic cancer prevention and therapy.

## Introduction

Pancreatic cancer is one of the most lethal cancers in the United States, ranking fourth among the leading causes of cancer-related deaths [1]. Despite treatment developments, the death rate for patients with pancreatic cancer has overall remained unchanged for decades. Investigations into novel therapies and chemopreventive agents are clearly warranted.

Studies have suggested that increased intake of dietary fruits, vegetables, and cereal grains may decrease pancreatic cancer risk [2], [3], [4]. Tocotrienols, found in cereal grains, comprise one of the most compelling groups of anti-tumor bioactive compounds [5]. Tocotrienols are a group of four (α-, β-, δ-, γ-) unsaturated, naturally occurring vitamin E compounds that not only inhibit the proliferation of a variety of human tumor cells, including breast, colon, lung, and hepatocellular [6], [7], [8], but also exhibit chemopreventive properties [9], [10]. However, how tocotrienols attenuate tumor proliferation is poorly understood.

We previously demonstrated that δ-tocotrienol exhibits the most potent anti-tumor activity among the four tocotrienol isoforms in pancreatic cancer cells [11], [12]. In an ongoing phase I dose-escalation clinical trial in pancreatic cancer patients, preliminary findings revealed that δ-tocotrienol had no obvious toxicity at up to 3200 mg/day, which is 5 times the predicted biologically active clinical dose [13]. These findings underscore the promise of δ-tocotrienol for pancreatic cancer intervention. To further translate these findings in the clinic, it is important to identify relevant biomarkers of δ-tocotrienol activity for early-phase hypotheses-driven clinical trials.

To this end, we investigated how δ-tocotrienol inhibits pancreatic cancer cell growth and identified the cyclin-dependent kinase (CDK) inhibitor p27^Kip1^ as a molecular target of δ-tocotrienol. p27^Kip1^ functions as a tumor suppressor by its ability to block cell proliferation. p27^Kip1^ is an atypical tumor suppressor because mutations of its gene are extremely rare. Nevertheless, tumor cells have evolved other mechanisms to inactivate p27^Kip1^, including increased proteolytic degradation and exclusion from the nucleus. In fact, p27^Kip1^ loss has been associated with pancreatic cancer progression and poor prognosis [14], [15], [16], [17]. Here, we report for the first time that p27^Kip1^ plays a central role in δ-tocotrienol-induced G_1_ arrest. We also observed that induction of p27^Kip1^ by δ-tocotrienol occurs at the transcription level involving E2F-1-mediated promoter activation and mRNA induction.

## Materials and Methods

### Chemicals

Purified δ-tocotrienol was initially supplied by Dr. Barry Tan (Hadley, MA) (90% δ-tocotrienol and 10% γ-tocotrienol; IC_50_: 15–20 µΜ) and subsequently by Davos Life Sciences (Singapore) (97% δ-tocotrienol; IC_50_: 50 µΜ) dissolved in ethanol as a stock solution and diluted to the required concentration with DMEM.

### Cell Lines and Culture

MIAPaCa-2, SW1990, and BxPC-3 pancreatic cancer cells were obtained from American Type Culture Collection (Manassas, VA) and grown to ∼70% confluency in DMEM supplemented with 10% FBS. HPDE6 C7, a human pancreatic duct epithelial cell line immortalized by transduction with E6/E7 genes of HPV-16 (generously provided by Dr. Ming-Sound Tsao, University of Toronto, Ontario, Canada [18]), was grown in serum-free keratinocyte medium as described previously [18]. Mouse embryonic fibroblasts (MEFs) having stable expression of p27^Kip1^ (+/+) and p27^Kip1^ (−/−) were provided by Dr. Pledger (Moffitt Cancer Center) [19], [20] and grown in DMEM with 10% FBS.

### Transfection and Generation of Stable Clones

MIAPaCa-2/shRNA p27^Kip1^ and MIAPaCa-2/vector were generated by transfecting MIAPaCa-2 cells with p27^Kip1^ shRNA already cloned into pSuperiorRetroPuro vector (OligoEngine, Seattle, WA), a kind gift from Dr. J. Chen (Moffitt Cancer Center) [21]. Stable puromycin-resistant clones were selected. Transfections were carried out with Metafectene (Biontex Laboratories, Planegg, Germany), per manufacturer's protocol.

### siRNA Knockdown of p27^Kip1^ in MIAPaCa-2 Cells

Pre-designed, siRNA to CDK inhibitor 1B (p27^Kip1^, #118714) and nonspecific siRNA (#4611) were purchased from Ambion (Austin, TX). MIAPaCa-2 cells were plated overnight in 12-well plates without antibiotic. Transient transfection of siRNA was carried out using Oligofectamine reagent (Invitrogen, Carlsbad, CA), per manufacturer's instructions. In brief, 5 nM p27^Kip1^ siRNA or control siRNA was mixed with Opti-MEM medium (Invitrogen) to a total volume of 90 µL and then complexed with 2 µL of Oligofectamine and 8 µL of Opti-MEM (total volume of complex was 100 µL). Before transfection, old medium was discarded, cells were washed with fresh Opti-MEM, and 400 µL of fresh Opti-MEM were placed into each well before adding the RNA-Oligofectamine complex. After 8 hours, 500 µL of DMEM containing 30% FBS and no antibiotics were added to each well, and cells were further incubated for 40 hours. After 40-hour transfection, cells were treated with δ-tocotrienol for an additional 24 hours and harvested for trypan blue and Western blot analysis.

### Protein Extraction and Western Blot Analysis

Cultured cells were lysed in mammalian protein extraction reagent (Pierce, Rockford, IL), per manufacturer's protocol. Antibody to p27^Kip1^ was purchased from BD Bioscience (San Jose, CA). Membranes were blocked in either 5% milk in PBS (pH 7.4) containing 0.1% Tween 20 or 1% bovine serum albumin (BSA) in TBS (pH 7.5) containing 0.1% Tween 20. Phospho-specific antibodies were incubated in 2% BSA in TBS (pH 7.5) containing 0.1% Tween 20; all other antibodies were diluted in 5% milk in PBS (pH 7.4) containing 0.1% Tween 20 overnight at 4°C. Horseradish peroxidase-conjugated secondary antibodies (Jackson ImmunoResearch, West Grove, PA) were diluted in 5% milk in either PBS (pH 7.4) containing 0.1% Tween 20 or TBS (pH 7.5) containing 0.1% Tween 20 at a 1∶1000 dilution for 1 hour at room temperature. Western blots were visualized using enhanced chemiluminescence (Pierce). We used beta-actin mouse monoclonal antibody (catalog #8H10D10) from Cell Signaling to ensure equal protein loading and expression.

### Anchorage-Independent Growth Assays

For soft agar growth assays, cells were seeded at 1×10^3^ cells/well in triplicate in 12-well culture dishes in 0.35% agar over a 0.6% agar layer. Various concentrations of δ-tocotrienol or vehicle were included in the 0.3% agar layer of cells. Cultures were fed and treated with compound or vehicle weekly until colonies grew to a suitable size for observation (∼3–4 weeks). Colonies were photographed after incubation with 1 mg/mL MTT overnight and counted. Growth of δ-tocotrienol-treated colonies was compared to vehicle-treated colonies (control). Three separate experiments were performed.

### FACS and Cell Proliferation Assay

Exponentially growing pancreatic cells were grown to 70% confluency in 96-well plates and incubated with increasing concentrations of δ-tocotrienol or vehicle for 24–72 hours. Wells were examined for cell growth and proliferation using the MTT colorimetric assay. IC_50_ results for each cell line were determined for each 24-hour time point. For cell-cycle analysis, pancreatic cells were grown to 70% confluency in 100-mm plates and then serum starved for 48 hours to allow for synchronization. After 48 hours, medium was aspirated and fresh medium with δ-tocotrienol (IC_50_) or vehicle was added for 24 hours. Treated medium was then collected, monolayers were washed with cold PBS, cells were trypsinized, and cell pellets were collected. Cell pellets were washed twice with PBS, fixed in cold methanol, and rewashed with PBS to remove methanol. After resuspension in 300–500 µL PBS, cells were digested with 20 µg/mL RNase and cellular DNA was stained with propidium iodide (50 µg/mL) by 3-hour incubation at room temperature in the dark. Cell-cycle distribution was analyzed by flow cytometry using a fluorescence-activated cell sorting (FACS) system (Becton Dickinson, Franklin Lakes, NJ).

### Confocal Microscopy Analysis

Treated MiaPaCa-2 cells (50,000) per 250 µL of 20% FBS-PBS were added to each cytofunnel slot and spun at 570 rpm for 5 minutes at high acceleration. Slides were removed and air dried at room temperature. Cells were fixed in 4% paraformaldehyde for 10 minutes, washed three times with 1X PBS with agitation for 5 minutes/wash, and then permeabilized in 0.5% Triton X-100 for 5 minutes at room temperature. Cells were blocked with 2% PBS-BSA (100 µL) for 30 minutes, and p27^Kip1^ antibody (1 ∶500) dilution prepared in 2% BSA was directly applied. Slides were incubated for 1 hour at room temperature in a closed humid chamber. Cells were then washed three times with 1X PBS with agitation for 5 minutes/wash. Secondary antibody (Alexa Fluor 594) in PBS (1∶500) was prepared and added to fixed cells. Slides were again incubated for 1 hour at room temperature in closed humid chamber and then washed three times with 1X PBS for 5 minutes/wash. VectaShield (50 µL) with DAPI was added, and coverslips were applied. After incubation at 4°C in dark conditions, slides were examined under a confocal microscope.

### Cytosolic and Nuclear Protein Extraction

Proteins were extracted using NE-PER Nuclear and Cytoplasmic Extraction Reagent Kit (Pierce). Treated MIAPaCa-2 cells were isolated as 20-µL packed cell volume (40 mg) in a 1.5-mL microcentrifuge tube by 5-minute centrifugation at 500×g. Supernatant was discarded, cell pellets were dried, and ice-cold CER-1 containing protease inhibitors (200 µL) was added. Tubes were vortexed vigorously for 15 seconds and incubated on ice for 10 minutes. Next, ice-cold CERII (11 µL) was added to tubes, which were vortexed for 5 seconds and incubated on ice for 1 minute, vortexed again for 5 seconds, and then centrifuged at 14,000×g for 5 minutes. Supernatant (cytosolic extract) was transferred to fresh pre-chilled tubes and stored at −80°C. We resuspended the insoluble pellet containing nuclei in 100 µL of ice-cold NER containing protease inhibitors. Tubes were vortexed for 15 seconds and kept on ice for 10 minutes. This step was repeated four times (40 minutes). Tubes were centrifuged at 14,000×g for 10 minutes, and supernatant (nuclear extract) was transferred to pre-chilled tubes and stored at −80°C.

### Luciferase Reporter Assay

MIAPaCa-2 cells were seeded in 6-well plates at 2×10^5^ cells/well. Each well was transfected the following day with 2 µg of p27^Kip1^ luciferase reporter plasmid (full-length p27^Kip1^-1609, different 5′ deletion mutants of mouse p27^Kip1^ promoter luciferase reporter, or pGL-3 base cDNA empty vector; all plasmids provided by Dr. Pledger) along with siRNA E2F-1 or non-target siRNA (Santa Cruz). Metafectene was used as the transfection reagent, per manufacturer's instructions. Lysates were collected 24 hours after δ-tocotrienol treatment (IC_50_ 50 µM). Luciferase activity was measured by the luciferase assay system kit (Promega). For normalization of transfection efficiency, 200 ng of Renilla reniformis luciferase expression plasmid (pRL-TK vector, Promega) was included in the transfection.

### RT-PCR Analysis

MIAPaCa-2 cells were seeded in 6-well plates and treated with δ-tocotrienol (IC_50_ 50 µM) or vehicle (as control) for 24 hours. Cells were then harvested in 1X lysis buffer, and RNA was isolated using Allprep RNA/protein kit according to manufacturer's instructions (Qiagen, Valencia, CA). Reverse transcription of total RNA was performed using the SuperScript III kit (Invitrogen). The following forward and reverse primers, respectively, were used for PCR reaction: for p27^Kip1^, 5′-TAACCCGGGACTTGGAGAAG and 5′-GCTTCTTGGGCGTCTGCTC to amplify a 450-bp product; for actin, 5′-GCTCGTCGTCGACAACGGCT and 5′-CAAACATGATCTGGGTCATCTTCTC to amplify a 353-bp product. To avoid overamplification, p27^Kip1^ mRNA expression levels were determined by RT-PCR at different amplification cycles (20, 25, 30, and 40 PCR cycles) and analyzed by agarose gel electrophoresis. Representative results at cycle 40 are shown.

### Cyclohexamide Blocks Protein Synthesis

After 12-hour δ-tocotrienol treatment (IC_50_ 50 µM), δ-tocotrienol was removed by rinsing MIAPaCa-2 cells three times with PBS; cyclohexamide (40 µg/mL) was then used to block protein synthesis. p27^Kip1^ turnover rate was examined by Western blot.

### Ethics Statement

This study was carried out in strict accordance with recommendations in the Guide for the Care and Use of Laboratory Animals of the National Institutes of Health. The protocol was approved by the University of South Florida Institutional Animal Care and Use Committee (Application 2805).

### Anti-tumor Activity in Nude Mouse Xenograft Model

We used female athymic nude (nu/nu) mice, 5–6 weeks old (Charles River, Wilmington, MA), for our animal studies. Animals were kept in clean cages limited to 4 mice per cage. The mice were cared for with ample food and water and examined on a daily basis. If tumors interfered with ambulation, caused signs of weight loss >10%, caused respiratory distress, or grew to >2 cm in size, the mice were humanely euthanized by exposure to increasing concentrations of carbon dioxide.

MIAPaCa-2 cells were harvested and resuspended in PBS (1×10^6^ cells/50 µL) and an equal volume of Matrigel (BD Biosciences). Cell samples (100 µL) were then injected subcutaneously into the right flank. Once tumors reached between 250 and 300 mm^3^, mice were randomized into groups of 10 and dosed by oral gavage with 0.1 mL of vehicle (purified olive oil) or δ-tocotrienol (100 mg/kg) daily for 3 weeks. Tumor volumes were determined twice per week by measuring length (*l*) and width (*w*) and by calculating the volume: V  =  (*l* + *w*)/2 × (*l* × *w*) × 0.5236. Statistical significance between control and treated animals was determined using Student's *t*-test.

### Immunohistochemistry and Slide Quantitation

Tumors from xenograft experiments were fixed in 4% paraformaldehyde, pH 7.2. After fixation, the tissue samples were processed into paraffin blocks. The primary antibodies used in this study were mouse monoclonal (p27, p-MAPK, and Ki-67) antibodies raised against the corresponding antigens of human origin in paraffin-embedded sections. Immunohistochemical staining was performed on a Ventana BenchMark XT (Tucson, AZ) automated slide stainer, using 4-µm thick paraffin sections from each of the representative tumor blocks selected. The sections were deparaffinized, rehydrated, and incubated with 3% H_2_O_2_ to block endogenous peroxidase. After antigen unmasking using proprietary CC1 solution for 60 minutes online (standard) at 100°C, the sections were incubated with antibodies to p27 (Kip 1, cloneSX53G8) (proprietary dilution, Cell Marque, Rocklin, CA) and Ki-67 (proprietary dilution, Ventana, Tucson, AZ). The incubation times were 32 minutes for p27 and Ki-67, according to the manufacturer's instructions. Phospho-p44/42 MAPK rabbit monoclonal antibody (ERK1/2)(Thr202/Tyr204) (catalog no. 4376, Cell Signaling, Danvers, MA) was used at a 1:200 concentration in PSS diluents (Ventana) and incubated for 32 minutes. The Ventana anti-rabbit secondary antibody was used for 20 minutes. The sections were then subjected to biotin block using a Ventana endogenous biotin kit (Ventana). The sections were incubated with biotin-labeled secondary antibody and streptavidin-peroxidase for 30 minutes each (DAKO Diagnostics). A solution of 3,3′-diaminobenzidene tetrahydrochloride (Sigma, St. Louis, MO) was used as a chromogen followed by sodium azide and 20 µL of H_2_0_2_ in 100 ml of Tris-HCl (50 µM, pH 7.6). After light counterstain with Harris' hematoxylin, the sections were examined under light microscopy.

### Evaluation of the Stains

The immunohistochemical expression of p27, p-MAPK, and Ki-67 proteins were determined as the product of immunostain intensity and percent of cells stained. These were scored on a 0-3 scale, with 3 being maximal. The immunostain intensity was scored with no staining being 0, light staining as 1, moderate staining as 2, and heavy staining as 3. The percent of cell stained was measured with no detectable staining as 0, 1–33% as 1, 34–66% as 2, and 67–100% as 3. The final IHC score was the product of the percent of cells stained score multiplied by the intensity score, allowing for a maximal score of 9 and a minimal score of 0.

### Statistical Analysis

Data, expressed as means ± SEM, were analyzed statistically using unpaired t-tests or one-way analysis of variance (ANOVA) where appropriate. We used GraphPad Prism version 5.04 for our analyses. Statistical significance was set at *P*<0.05.

## Results

### δ-Tocotrienol Inhibits Anchorage-Dependent and –Independent Cell Growth and Induces G_1_ Arrest in Pancreatic Cancer Cells

**Figure 1:**
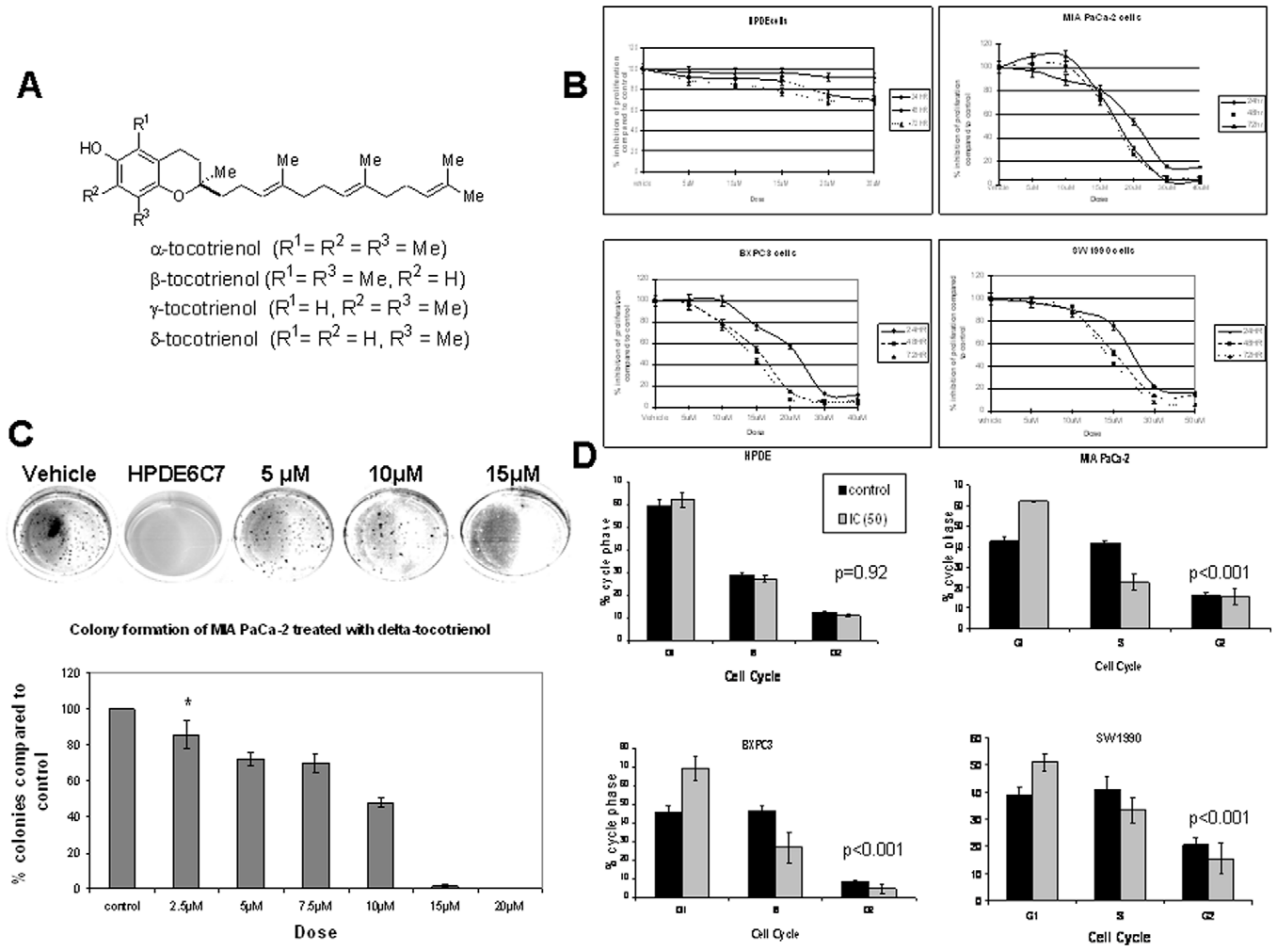
δ-Tocotrienol inhibits pancreatic cancer growth. **A**, Chemical structures of tocotrienols. **B**, δ-tocotrienol selectively inhibits pancreatic cancer cell proliferation. HPDE6 C7 and human pancreatic cancer cell lines MIAPaCa2, BXPC3, and SW1990 were treated with increasing concentrations of δ-tocotrienol or vehicle and analyzed by MTT at 24, 48, and 72 hours. Results demonstrate selective inhibition of pancreatic cancer cells in a time- and dose-dependent manner. **C**, δ-tocotrienol inhibits anchorage-independent growth of transformed MIAPaCa-2 pancreatic cancer cells. Number of colonies was normalized and compared to vehicle. *Concentration at which statistical significance begins. **D**, effects of δ-tocotrienol on cell cycle progression. Pancreatic cancer cells and HPDE6 C7 cells were incubated in the presence of δ-tocotrienol (IC_50_) (gray) or vehicle (black) as control for 24 hours. Cells were collected for determination of cell cycle distribution by FACS analysis after cell staining with propidium iodide. Each analysis represents means ± SD of 3 independent experiments. δ-Tocotrienol inhibits G_1_-to-S cell cycle progression selectively in transformed pancreatic cancer cell lines.

The effects of δ-tocotrienol ( 1A) on cell growth in MIAPaCa-2, BxPC-3, and SW1990 human pancreatic cancer cells were examined by 3-(4,5-dimethylthiazol-2-yl)-2,5-diphenyltetrazolium bromide (MTT) cell proliferation assay. Cells were treated with 0-50 µM δ-tocotrienol for 24, 48, and 72 hours. The effects of δ-tocotrienol were also evaluated in the non-transformed human pancreatic ductal epithelial cell line HPDE6 C7 to rule out possible cytotoxic effects of δ-tocotrienol on non-neoplastic cells. δ-Tocotrienol treatment inhibited anchorage-dependent cell proliferation in both a time- and concentration-dependent manner in human pancreatic cancer cells (Figure 1B); however, no significant growth inhibitory effects were noted in HPDE6 C7 cells. δ-Tocotrienol treatment also significantly inhibited colony formation in MIAPaCa-2 cells grown in soft agar from 2.5 µM (*P* = 0.02) (Figure 1C).

δ-Tocotrienol treatment also had a significant selective effect on cell-cycle progression, as demonstrated by an increase in the percentage of pancreatic cancer cells but not HPDE6 C7 cells in the G_1_ phase (*P*<0.001 vs. *P* = 0.92) (Figure 1D). We found that the pancreatic cancer cells accumulated in the G_1_ phase at the expense of a decrease in the S-phase population.

### δ-Tocotrienol Induces p27^Kip1^ Expression and Inhibits RAS-MEK-ERK Signaling

**Figure 2:**
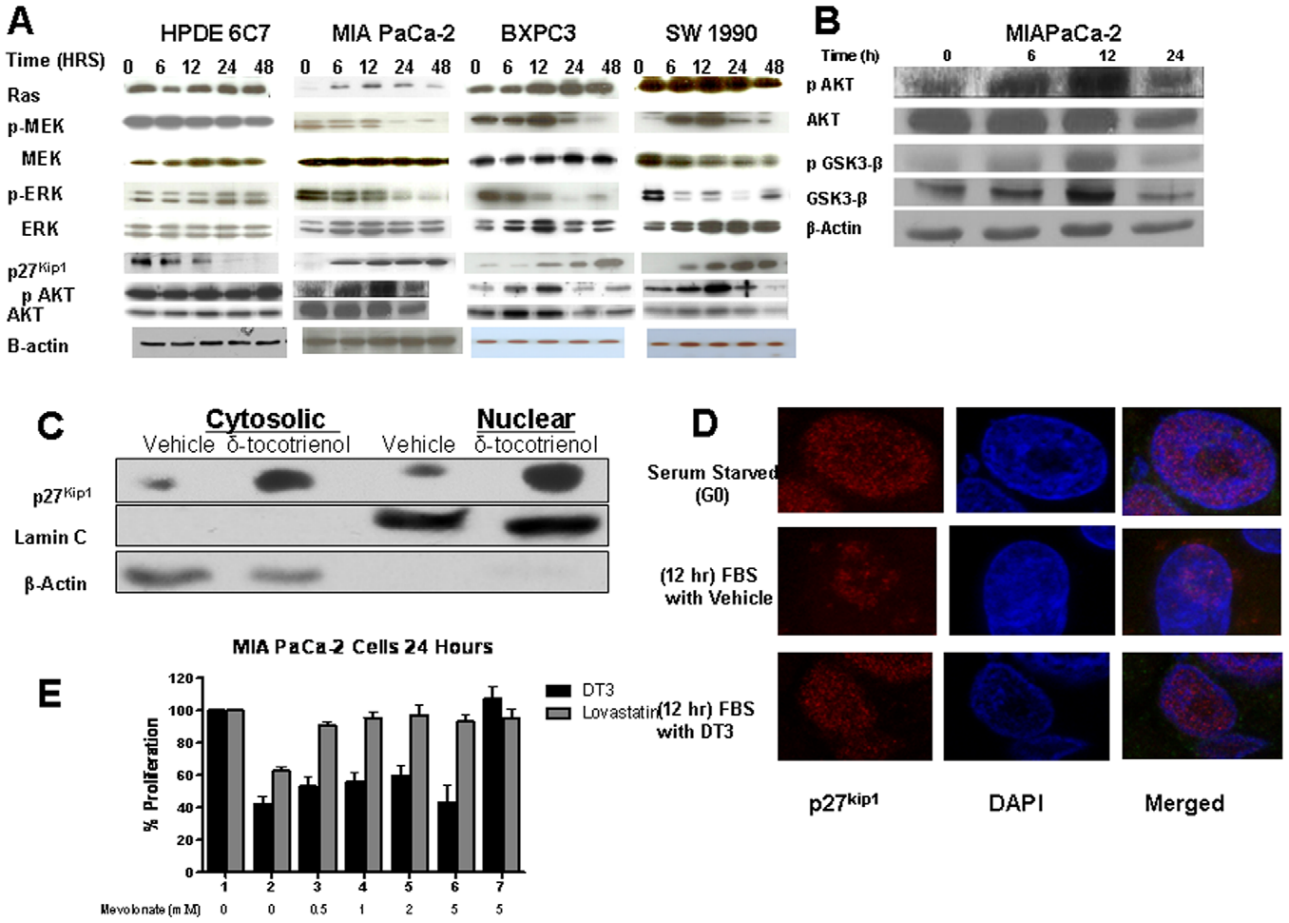
δ-Tocotrienol induces p27^Kip1^ expression and inhibits RAS-MEK-ERK signaling. **A**, pancreatic cancer and HPDE6 C7 cells were treated with δ-tocotrienol at predetermined IC_50_ for each cell line. Protein lysates from 0–48 hours were collected and analyzed by Western blot analysis for Ras oncogenic signaling targets, MEK and ERK, and p27^Kip1^. Results are representative of 3 independent experiments. δ-Tocotrienol selectively inhibits MAP kinase signaling and increases p27^Kip1^ expression in transformed pancreatic cancer cells. **B,** MIAPaCa-2 cells were treated with δ-tocotrienol at predetermined IC_50_. Protein lysates from 0–24 hours were collected and analyzed by Western blot for AKT and a downstream target GSK-3β. **C**, MIAPaCa-2 cells were treated with vehicle or δ-tocotrienol in FBS for 12 hours, and pure nuclear and cytosolic fractions were isolated. Western blots show increased levels of p27^Kip1^ in δ-tocotrienol treated cells with high concentrations in the nucleus. **D,** simultaneously, whole cells were stained with immunofluorescent p27^Kip1^ antibody and analyzed by fluorescent microscopy for p27^Kip1^ localization. δ-Tocotrienol localized p27^Kip1^ to the nucleus similar to the starved state, whereas serum-treated cells showed equal levels of p27^Kip1^ in the nucleus and cytoplasm. **E,** δ-tocotrienol (DT3) suppresses tumor cell growth of MIAPaCa-2 cells in the presence of added mevalonate, a metabolite of HMG-CoA reductase. MIAPaCa-2 cells (in 96-well plates) were treated for 24 hours with δ-tocotrienol or lovastatin in the absence or presence of increasing concentrations of mevalonate. MTT results demonstrate that mevalonate rescues the growth inhibitory effects of lovastatin, but not that of δ-tocotrienol. In **E**, lines 1 and 7 had no δ-tocotrienol (DT3) or lovastatin.

Regulation of intracellular signaling pathways is central to the ability of oncogenes to promote cell-cycle progression. Two major pathways intimately involved in the G_1_-to-S traverse are the RAS-activated RAF-MEK-ERK and PI3-AKT pathways. These pathways influence the expression, activity, or subcellular localization of key components of the cell-cycle machinery such as cyclins, CDKs, and CDK inhibitors, leading to the appropriate activation of E2F-1 transcription factors. Several agents have been described that regulate the G_1_ traverse and transition into the S-phase in pancreatic cancer cells, and p27^Kip1^ has been reported to be increased by these agents [22], [23], [24], [25], [26]. We therefore determined the kinetics of p27^Kip1^ levels and RAF-MEK-ERK pathway activity in pancreatic cancer cells and in HPDE6 C7 cells exposed to δ-tocotrienol. We found that δ-tocotrienol significantly increased p27^Kip1^ levels by 6 hours in the pancreatic cancer cells (Figure 2A). This increased level was sustained and increased in all cell lines by 48 hours and associated with corresponding inhibition of the activity of the activated RAF-MEK-ERK signaling pathway, as measured by decreased phosphorylated MEK and ERK levels in the pancreatic cancer cells (Figure 2A). In contrast, the PI3-AKT pathway was initially induced at 12 hours followed by subsequent inhibition at 24 hours, as measured by phosphorylated AKT levels (Figure 2A and 2B). Consistent with the effect on proliferation, δ-tocotrienol suppressed p27^Kip1^ levels in the nontransformed human pancreatic ductal cell line, HPDE6 C7, and had no effect on MEK, ERK, or AKT. We also showed that the effects of δ-tocotrienol were specific to malignant cells, as δ-tocotrienol did not induce p27^Kip1^ levels or alter MEK, ERK, or AKT expression in the nontransformed pancreatic ductal epithelial cells. The mechanisms of this need further investigation.

Besides modulation of its expression levels, subcellular localization is also important in governing p27^Kip1^ function. To act as a cell-cycle inhibitor, p27^Kip1^ must be located in the nucleus, whereas its cytoplasmic mislocalization favors cell-cycle progression and may contribute to cellular transformation [27], [28]. Figure 2C shows that p27^Kip1^ levels are increased in both the cytosolic and nuclear compartments of δ-tocotrienol-treated MIAPaCa-2 cells compared to vehicle. In Figure 2D, we observed p27^Kip1^ cytoplasmic accumulation in vehicle-treated MIAPaCa-2 cells versus increased p27^Kip1^ levels in the nuclear compartment of serum-starved quiescent MiaPaCa-2 cells and δ-tocotrienol-treated MIAPaCa-2 cells. Some authors have suggested that tocotrienols modulate HMG-CoA reductase activity via post-transcriptional actions, thereby inhibiting farnesylation of downstream oncogenic signaling targets, similar to the effects of lovastatin [29], [30], [31]. To determine whether δ-tocotrienol inhibits proliferation through mevalonate pathway inhibition, we treated MIAPaCa-2 cells with δ-tocotrienol or lovastatin and with increasing concentrations of mevalonate, a downstream product of HMG-CoA reductase. Addition of mevalonate to δ-tocotrienol-treated cells resulted in no significant rescue from the growth inhibitory effects of δ-tocotrienol on MIAPaCa-2 cells (Figure 2E); however, lovastatin's ability to inhibit proliferation was rescued by mevalonate.

### p27^Kip1^ Is Required for δ-Tocotrienol-Induced G_1_ Arrest

**Figure 3:**
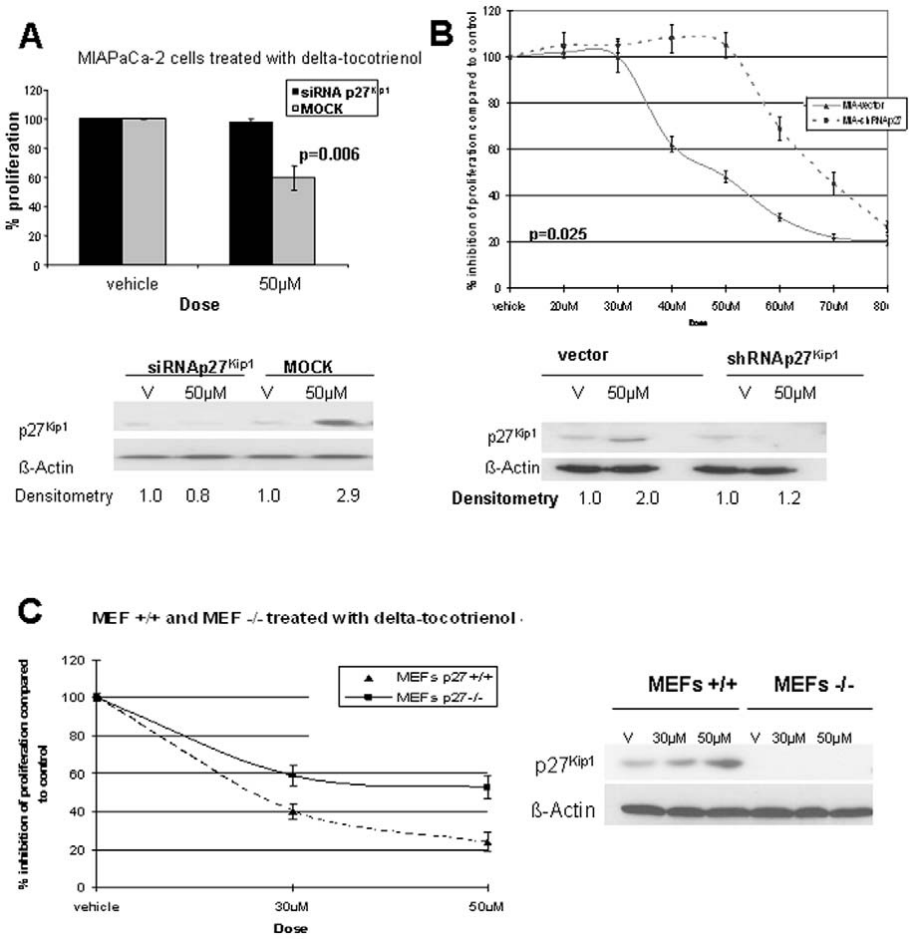
p27^Kip1^ is required for δ-tocotrienol-induced G_1_ arrest. **A,** p27^Kip1^ siRNA attenuates δ-tocotrienol-mediated growth suppression in human MIAPaCa-2 pancreatic cancer cells. After transfection with p27^Kip1^ siRNA or with noncoding siRNA for 24 hours, MIAPaCa-2 cells were incubated with fresh medium containing either δ-tocotrienol (IC_50_) or vehicle for an additional 24 hours. Cells were taken from culture and divided into 2 aliquots. Immunoblots demonstrate inhibited p27^Kip1^ expression with siRNA p27^Kip1^ and rescue from inhibition of proliferation in siRNA p27^Kip1^-pretreated cells. **B,** MIAPaCa-2 cells expressing stable shRNA p27^Kip1^ are protected from the growth inhibitory effects of δ-tocotrienol. Stable MIAPaCa-2 cells expressing shRNAp27^Kip1^ or empty vector were treated with increasing concentrations of δ-tocotrienol or vehicle for 24 hours, with proliferation determined by MTT assay. MIAPaCa-2 cells expressing shRNAp27^Kip1^ demonstrate resistance to growth inhibitory effects of δ-tocotrienol. **C,** p27^Kip1^ knockout cells attenuate δ-tocotrienol-mediated growth inhibitory effects. Stable MEF p27^Kip1^ (−/−) and MEF p27^Kip1^ (+/+) were plated and incubated with either δ-tocotrienol at the indicated doses or with vehicle for 48 hours. Cell cultures were then collected in 2 aliquots and analyzed as reported previously for siRNA p27^Kip1^-treated cells. In the absence of p27^Kip1^ expression, δ-tocotrienol exerts minimal growth inhibitory effects in mouse epithelial cells.

To determine the role of p27^Kip1^ induction in δ-tocotrienol pancreatic cancer cell growth inhibition, we transiently reduced p27^Kip1^ expression in MIAPaCa-2 cells using p27^Kip1^ small interfering RNA (siRNA; confirmed by Western blot). At 24 hours, absence of p27^Kip1^ expression significantly abrogated δ-tocotrienol-induced cell growth inhibition (Figure 3A). To confirm these findings, stable cell lines expressing p27^Kip1^ short hairpin RNA (shRNA) or vector were created using MIAPaCa-2 cells. Reduced p27^Kip1^ expression was confirmed by Western blot analysis. Figure 3B shows that depletion of p27^Kip1^ resulted in resistance to δ-tocotrienol treatment. To investigate whether these observations could be generalized beyond MIAPaCa-2 cells, we analyzed stable MEFs [19], [20] exhibiting p27^Kip1^ knock-out or wild-type cells after δ-tocotrienol treatment. We found that the lack of p27^Kip1^ rendered MEFs partially resistant to the anti-proliferative effects of δ-tocotrienol in this cell line (Figure 3C). Together, these studies underline the importance of p27^Kip1^ in δ-tocotrienol-induced G_1_ arrest and show that the contribution of this CDK inhibitor is not cell line specific.

### δ-Tocotrienol Regulates p27^Kip1^ Transcription in an E2F-1-Dependent Manner

**Figure 4:**
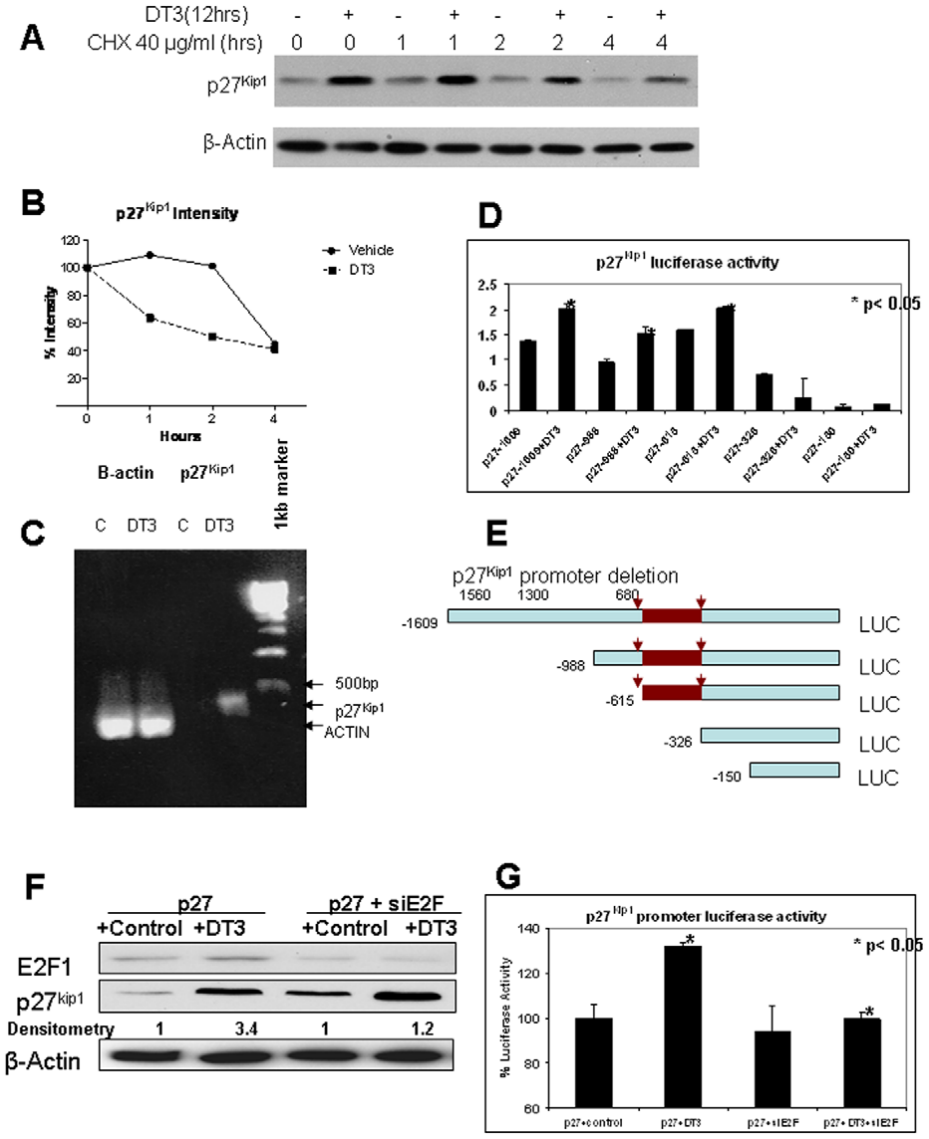
δ-Tocotrienol (DT3) regulation of p27^Kip1^ protein expression at the transcription level. **A,** MIAPaca-2 cells were treated with δ-tocotrienol at a predetermined IC_50_ and then with cyclohexamide to block protein synthesis. p27^Kip1^ turnover rate was examined by Western blot. **B,** densitometry plot for panel **A** using β-actin for density control, showing similar rates of degradation by δ-tocotrienol. **C,** RT-PCR confirms upregulation of p27^Kip1^ at the mRNA level. MIAPaCa-2 cells were treated with δ-tocotrienol for 24 hours, and p27^Kip1^ mRNA expression level was determined by RT-PCR at different amplification cycles. Representative result at cycle 40 is shown. **D,** activation of p27^Kip1^ promoter by δ-tocotrienol. MIAPaCa-2 cells were transfected with 5′-deletion mutants of the mouse p27^Kip1^ promoter luciferase reporter. After 24-hour transfection, cells were treated with δ-tocotrienol (IC_50_) or vehicle for an additional 24 hours, and luciferase activity was determined. Deletion analysis of the mouse p27^Kip1^ promoter suggests that the region between 326 and 615 contains sequences necessary for significant response to δ-tocotrienol in p27^Kip1^ reporter assays. **E,** illustration of 5′ deletion mutants of the mouse p27^Kip1^ promoter luciferase reporter. Sequence search of this region revealed several putative E2F-1 binding sites (TTTGGCTA, GCGCGGAG, GCGCCGAG) as demonstrated in the shaded area of the deletion mutant constructs. **F**, immunoblot showing suppressed E2F-1 expression using siRNA E2F-1, **G**, attenuated δ-tocotrienol mediated effects on the transfected full-length p27^Kip1^ promoter activity using a luciferase reporter assay.

CDK inhibitor levels are intricately regulated at the level of mRNA transcription/protein synthesis and/or degradation. To determine the effects of δ-tocotrienol on p27^Kip1^ protein stability, we conducted cyclohexamide chase experiments. As shown in Figure 4A and 4B, the pattern of p27^Kip1^ degradation in δ-tocotrienol-treated cells was similar to corresponding controls. Protein degradation at 4 hours in vehicle-treated cells was 45% versus 41% in δ-tocotrienol-treated cells. These results indicate that mechanisms involved in δ-tocotrienol-induced p27^Kip1^ expression were not due to protein stabilization.

We next investigated whether δ-tocotrienol induced p27^Kip1^ expression at the transcriptional level. Real-time PCR showed that δ-tocotrienol treatment significantly increased p27^Kip1^ mRNA levels compared to vehicle in MIAPaCa-2 cells, suggesting that the δ-tocotrienol-induced increase in p27^Kip1^ protein levels is through induction of p27^Kip1^/mRNA transcription (Figure 4C). δ-Tocotrienol's effect on p27^Kip1^ transcription was confirmed with promoter reporter assays. δ-Tocotrienol treatment significantly induced (∼50%) -1609 p27^Kip1^ promoter activity, demonstrating that δ-tocotrienol regulates p27^Kip1^ protein expression at the transcription level. Moreover δ-tocotrienol-dependent p27^Kip1^ promoter activation was maintained upon 5′-deletion to -615 bp but not upon further deletion to -326 bp. The -615/-326 DNA region contains three E2F-1 binding sites, which we have previously identified as crucial elements for the regulation of this gene (Figure 4D and 4E) [21]. To determine whether δ-tocotrienol E2F-1 binding to the p27^Kip1^ promoter was important in δ-tocotrienol-induced p27^Kip1^ transcription, we performed Western blot (Figure 4F) and reporter assays (Figure 4G) with knock-down of E2F-1 protein. As shown in Figure 4G, suppression of E2F-1 expression significantly inhibited δ-tocotrienol's ability to induce p27^Kip1^.

### Effects of δ-Tocotrienol on Pancreatic Tumor Cell Growth *In Vivo*

**Figure 5:**
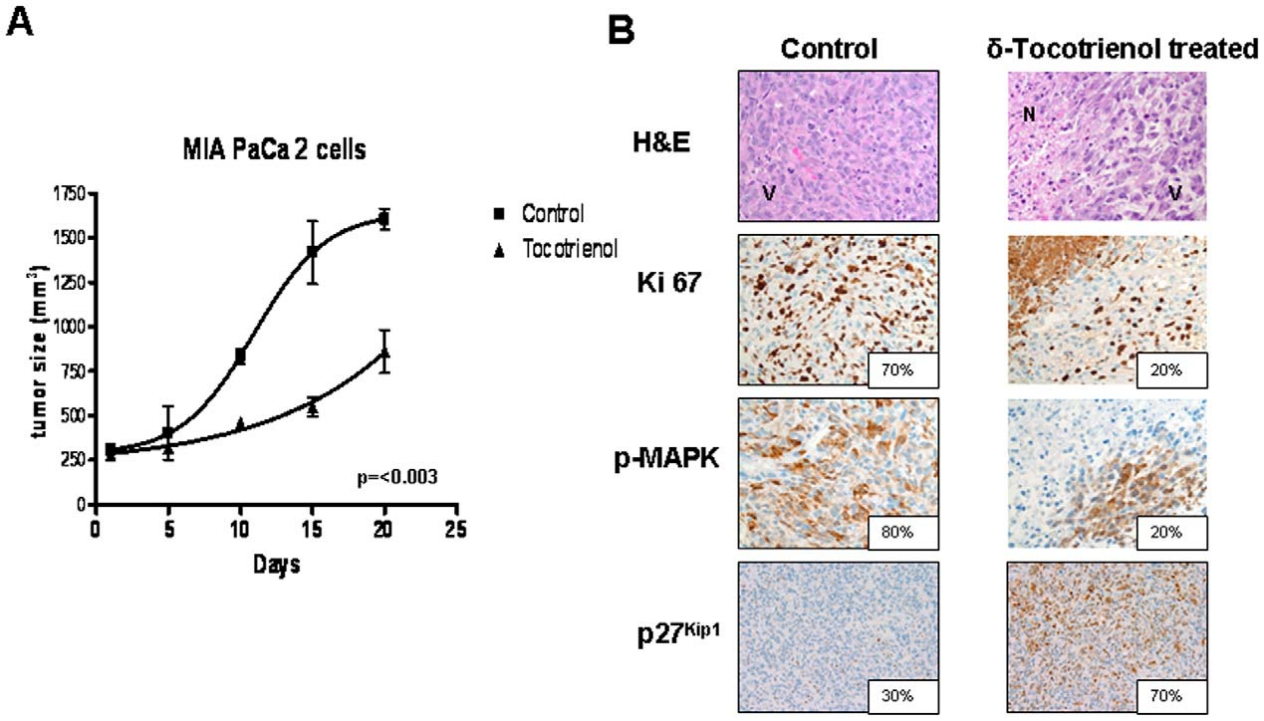
δ-Tocotrienol suppresses pancreatic tumor growth in nude mice. **A,** nu/nu mice were injected subcutaneously with a suspension of MIAPaCa-2 cells (1×10^6^) combined with Matrigel in both flanks. Tumor volume was recorded every other day according to the formula V  =  (*l* + *w*)/2 × (*l* × *w*) × 0.5236, where *l* is length and w is width. Tumors with equal growth rates and initial volumes between 250 and 300 mm^3^ were then randomized to receive δ-tocotrienol (100 mg/kg) or vehicle (purified olive oil) daily by gavage. Data showing significant inhibition of tumor growth by δ-tocotrienol are representative of 5 tumors/treatment group repeated on 3 separate occasions (*P*<0.003). **B,** tissue sections from tumors were fixed in paraformaldehyde and embedded in paraffin for immunohistochemical staining of Ki67, MAP kinase, and p27^Kip1^. Representative sections are shown. Areas of tumor necrosis (N) were consistently visualized in the δ-tocotrienol group in contrast to the vehicle group where only viable (V) tumor was present. H&E, hematoxylin and eosin. Numbers represent percentage of positive cells. H&E and immunohistochemistry, x200.

Using nude mouse xenografts, we found significant inhibition of tumor growth in mice treated with δ-tocotrienol (Figure 5A). Furthermore, immunohistochemical analysis demonstrated decreased Ki67, confirming inhibition of proliferation, decreased phosphorylated MAPK expression, and induced expression of p27^Kip1^ (Figure 5B). These findings correlate with our *in vitro* results and support the concept that Ki67 and p27^Kip1^ expression levels are important potential biomarkers in δ-tocotrienol treatment of pancreatic cancer.

## Discussion

The novel and central finding in this study is that p27^Kip1^ is indispensable in causing growth arrest in human pancreatic cancer cells by δ-tocotrienol *in vitro*. In this study, MEFs [19], [20] in which p27^Kip1^ was knocked out and human pancreatic cancer MIAPaCa-2 cell variants in which p27^Kip1^ was depleted were resistant to δ-tocotrienol-induced cell growth inhibition. Furthermore, δ-tocotrienol up-regulation of p27^Kip1^ expression in MIAPaCa-2 cells was through induction of E2F-1-dependent p27^Kip1^ transcription. These results establish increased p27^Kip1^ levels as a significant molecular biomarker of δ-tocotrienol anti-tumor efficacy against pancreatic cancer.

A key property of cancer cells and their progeny is their ability to reproduce in defiance of the normal restraints on cell division [32]. Limitless replicative potential is a hallmark of pancreatic oncogenesis. The mechanism of limitless replicative potential is linked to G_1_-to-S cell-cycle progression because this cell-cycle phase is linked to external stimuli and also commits the cell to DNA replication and subsequent mitosis. G_1_ checkpoint abrogation is a common phenomenon in carcinogenesis, giving tumor cells limitless replicative potential. In animal cells, entry of cells into a new cell cycle is controlled by regulation of CDK activity in G_1_. One of the critical mechanisms by which animal cells suppress CDK activity in G_1_ is through accumulation of the CDK inhibitor p27^Kip1^. p27^Kip1^ physically interacts with CDK via its amino-terminal domain, inhibiting CDK activity. Thus, activating the G_1_ checkpoint by upregulating the expression of p27^Kip1^ is a logical approach for controlling cancer cell proliferation. In this study, we showed that p27^Kip1^ induction is a crucial event in δ-tocotrienol-induced G_1_ arrest and inhibition of cell proliferation in MIAPaCa-2 pancreatic cancer cells. More importantly, this study provides evidence that p27^Kip1^ can function in the G_1_-to-S transition checkpoint and mediate δ-tocotrienol-induced G_1_ arrest and cell growth inhibition in both MIAPaCa-2 pancreatic cancer cells and MEFs.

Once we identified that up-regulation of p27^Kip1^ plays a central role in δ-tocotrienol-induced G_1_ arrest, our next aim was to identify the mechanism by which δ-tocotrienol induced p27^Kip1^ expression. p27^Kip1^ can have opposing roles in the process of malignant transformation, dependent on its cellular localization. When localized in the nucleus, it functions as a tumor suppressor by acting as a negative regulator of the G_1_-S transition by binding and inhibiting the cyclin E-CDK2 complex. Cytoplasmic localization of p27^Kip1^ supports the assembly and nuclear import of cyclin D-CDK4/6, thus promoting cell proliferation. Moreover, cytoplasmic mislocalization of p27^Kip1^ seems to contribute to the progression of many cancers by increasing cell motility and metastases and, importantly, inhibiting apoptosis [33], [34]. Therefore, p27^Kip1^ can be considered a nuclear tumor suppressor and a cytoplasmic oncogene. In evaluating whether the δ-tocotrienol-induced increase in p27^Kip1^ levels in pancreatic cancer cells occurred in a specific cellular compartment, we observed increased p27^Kip1^ in the nucleus of pancreatic cancer cells in vitro and in vivo. These findings suggest that δ-tocotrienol treatment favors the tumor-suppressive function of p27^Kip1^.

Ras activation indirectly causes cytoplasmic localization of p27^Kip^ via activation of its effector pathways, such as RAF-MEK-ERK. Phosphorylation of p27^Kip1^ on 3 different sites (Ser-10, Thr-157, and Thr-198) by components of these signaling pathways has been shown to result in p27^Kip1^ cytoplasmic localization [33], [35], [36], [37], [38], [39], [40], [41], [42]. Ras-regulated signaling pathways play an important role in initiation and progression of human pancreatic cancer [43], [44], [45]. In particular, the Ras→RAF-MAP-ERK kinase (MEK)→ERK-MAPK pathway plays an important regulatory role in cell-cycle division [46], [47], [48]. Gysin et al. showed that pharmacologic inhibition of RAF-MEK-ERK signaling in pancreatic cancer cells inhibits cell growth and results in G_1_ cell-cycle arrest through induced expression of p27^Kip1^ [49]. Our results show that p27^Kip1^ induction by δ-tocotrienol is related to inhibition of the RAF-MEK-ERK pathway. The mechanism by which δ-tocotrienol inhibits Ras activation signaling is poorly understood. A previous study implicated δ-tocotrienol inhibition of Ras prenylation by its suppression of HMG-CoA activity [50]. We did not observe any rescue of δ-tocotrienol anti-proliferative activity in MIAPaCa-2 cells with mevalonate supplementation, suggesting that δ-tocotrienol inhibition of activated Ras effector signaling is not by inhibition of Ras prenylation through inhibition of HMG-CoA reductase.

Mechanisms by which tumor cells inactivate p27^Kip1^ include increased proteolytic degradation and inhibition of transcription. Our results demonstrated that δ-tocotrienol did not affect the proteolytic degradation compared to control at 4 hours, but induced the transcription of p27^Kip1^. However, the effects of δ-tocotrienol on p27^Kip1^ promoter activity are modest, indicating that transcriptional stimulation might not be the sole contributor to δ-tocotrienol-induced p27^Kip1^ mRNA accumulation. Additional post-transcriptional mechanisms such as message stabilization by RNA-binding proteins might also be involved. Using p27^Kip1^ promoter-deletion mutants and E2F-1 genetic knockdown, we demonstrated that δ-tocotrienol-induced p27^Kip1^ promoter activity involves E2F-1. At first glance, this induction appears paradoxical because the main function of p27^Kip1^ is to inhibit E2F-1 activation by inhibiting the phosphorylation of retinoblastoma protein through inhibition of the G_1_ CDK. However, induction of p27^Kip1^ expression by E2F-1 may potentially function as a feedback regulatory mechanism that limits E2F-1 activity [21]. We have shown that suppression of E2F-1 expression by siRNA correlated with significantly reduced p27^Kip1^ levels and, alternatively, inhibition of p27^Kip1^ expression by siRNA-enhanced E2F-1 function [21]. Together, our findings suggest that δ-tocotrienol may initially promote E2F-1 function early in the cell's exposure, resulting in E2F-1 binding to the p27^Kip1^ promoter and up-regulation of p27^Kip1^ transcription and protein expression. Increased p27^Kip1^ protein levels then inhibit G_1_ CDK, which inhibits Rb-phosphorylation and thereby E2F-1 activation.

δ-Tocotrienol has shown promising efficacy against several cancer models, including pancreatic cancer [11], [12], [50]. We have almost completed a phase I dose-escalation study of patients with pancreatic cancer. To advance the development of δ-tocotrienol into phase II clinical trials, the molecular targets that play a central role in δ-tocotrienol efficacy in pancreatic cancer need to be elaborated so that they can be used as biomarkers of p27^Kip1^ efficacy. Here, we show convincingly for the first time that up-regulation of p27^Kip1^ is an important event in δ-tocotrienol-induced G_1_ arrest in human pancreatic cancer cells. In summary, our study provides the experimental justification for the use of nuclear p27^Kip1^ as molecular markers of δ-tocotrienol efficacy in human pancreatic tumor.
